# Synthesis and Evaluation of Aminothiazole-Paeonol Derivatives as Potential Anticancer Agents

**DOI:** 10.3390/molecules21020145

**Published:** 2016-01-26

**Authors:** Chia-Ying Tsai, Mohit Kapoor, Ying-Pei Huang, Hui-Hsien Lin, Yu-Chuan Liang, Yu-Ling Lin, Su-Chin Huang, Wei-Neng Liao, Jen-Kun Chen, Jer-Shing Huang, Ming-Hua Hsu

**Affiliations:** 1 Nuclear Science & Technology Development Center, National Tsing Hua University, Hsinchu 30013, Taiwan; s101000017@m101.nthu.edu.tw; 2 Department of Chemistry, National Tsing Hua University, Hsinchu 30013, Taiwan; mohitk48@gmail.com (M.K.); suzann800217@hotmail.com (Y.-P.H.); jshuang@mx.nthu.edu.tw (J.-S.H.); 3 Division of Radiotherapy, Department of Oncology, Taipei Veterans General Hospital, Taipei 11217, Taiwan; twwarcgogo@gmail.com; 4 Agricultural Biotechnology Research Center, Academia Sinica, Taipei 115, Taiwan; ycliang@sinica.edu.tw; 5 Department of Biological Science and Technology, National Chiao Tung University, Hsinchu 30010, Taiwan; lyring@pchome.com.tw; 6 Center for Bioinformatics Research, National Chiao Tung University, Hsinchu 30010, Taiwan; 7 Institute of Biomedical Engineering and Nanomedicine, National Health Research Institutes, Miaoli 35053, Taiwan; chin@nhri.org.tw (S.-C.H.); wei-neng@nhri.org.tw (W.-N.L.)

**Keywords:** paeonol, 2-aminothiazole, anti-cancer, sulfonate, adenocarcenoma

## Abstract

In this study, novel aminothiazole-paeonol derivatives were synthesized and characterized using ^1^H-NMR, ^13^C-NMR, IR, mass spectroscopy, and high performance liquid chromatography. All the new synthesized compounds were evaluated according to their anticancer effect on seven cancer cell lines. The experimental results indicated that these compounds possess high anticancer potential regarding human gastric adenocarcinoma (AGS cells) and human colorectal adenocarcinoma (HT-29 cells). Among these compounds, *N*-[4-(2-hydroxy-4-methoxyphenyl)thiazol-2-yl]-4-methoxybenzenesulfonamide (**13c**) had the most potent inhibitory activity, with IC_50_ values of 4.0 µM to AGS, 4.4 µM to HT-29 cells and 5.8 µM to HeLa cells. The 4-fluoro-*N*-[4-(2-hydroxy-4-methoxyphenyl)thiazol-2-yl]benzenesulfonamide (**13d**) was the second potent compound, showing IC_50_ values of 7.2, 11.2 and 13.8 µM to AGS , HT-29 and HeLa cells, respectively. These compounds are superior to 5-fluorouracil (5-FU) for relatively higher potency against AGS and HT-29 human cancer cell lines along with lower cytotoxicity to fibroblasts. Novel aminothiazole-paeonol derivatives in this work might be a series of promising lead compounds to develop anticancer agents for treating gastrointestinal adenocarcinoma.

## 1. Introduction

**Figure 1:**
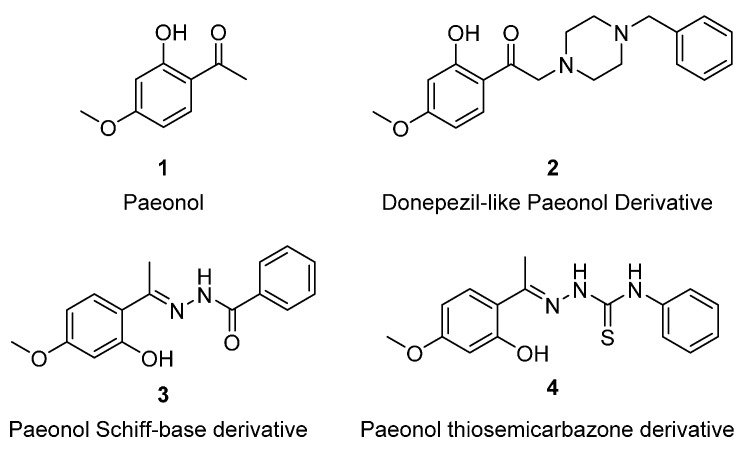
Structures of paeonol, donepezil-like paeonol derivative, paeonol Schiff-base derivative, and paeonol thiosemicarbazone derivative.

Paeonol, 2-hydroxy-4-methoxy acetophenone (**1**, Figure 1), is a major component of traditional Chinese medicine. Moutan Cortex, the outer layer of the root of Moutan, is classified in the genus *Paeonia* and has been used for more than 1000 years. Paeonol is categorized as a flavonoid derivative and exhibits many remarkable biological effects, and it has been applied for anti-inflammatory [1,2], analgesic [2], antioxidant [3], antidiabetic [4], anticancer [5], and antiatherogenic purposes [6]. Paeonol was also found to protect against memory loss after ischemic stroke by reducing amyloid precursor protein (APP), beta-site APP cleaving enzyme (BACE), and apoptosis [7]. Moreover, paeonol derivatives have been reported to show many attractive biological activities. For example, Pan and Hui demonstrated that the donepezil-like paeonol derivative (**2**, Figure 1) exhibited strong metal-chelating ability for Alzheimer’s disease (AD) treatment [8]. Yang presented a copper ion chelating paeonol Schiff-base derivative (**3**, Figure 1) complexes that possessed high antioxidant activity and moderate DNA-binding activity as well as high tumor cell cytotoxicity [9]. Moreover, Yu reported a paeonol thiosemicarbazone derivative (**4**, Figure 1), which exhibited potential mushroom tyrosinase inhibitors [10]. Recently, our group found that phenylsulfonyl moieties-conjugated paeonol derivatives were potential anti-Hepatitis B virus leads [11] and could prevent lipid accumulation at lower doses, and they might be prominent antiatherogenic agents [12].

**Figure 2:**
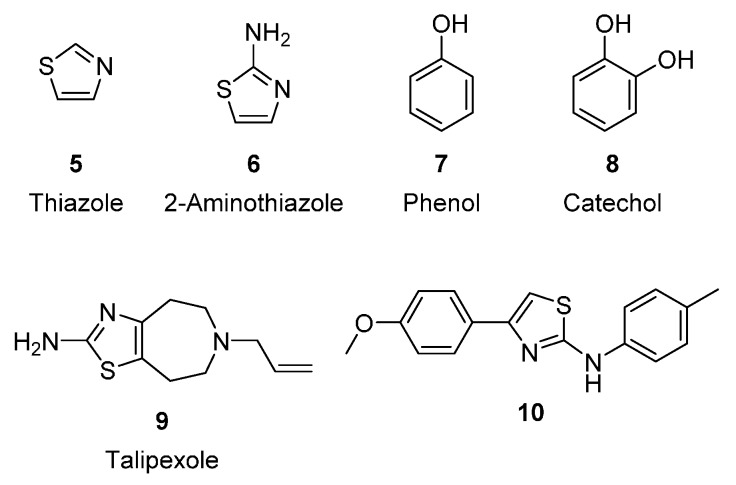
Structures of thiazole, 2-aminothiazole, phenol, catechol, talipexole and 2-aminothiazole derivative.

The thiazole ring (**5**, Figure 2), a five-membered heterocyclic core structure, displays a variety of biological effects, such as antibacterial, antifungal, anti-Human immunodeficiency virus, anti-inflammatory, antidiabetic, antioxidant, and anticancer effects [13]. These heterocyclic rings, notably 2-aminothiazole (**6**, Figure 2), are considered stable and lipophilic bioisosteres of phenol (**7**, Figure 2) or catechol (**8**, Figure 2) moieties, which might retain pharmacological action while having improved oral bioavailability [14]. Talipexole (**9**, Figure 2), a dopamine agonist for Parkinson’s disease treatment, was designed on the basis of the bioisosteric effect of phenol and 2-aminothiazole [15]. In addition, the 2-aminothiazole core was found to act as the pharmacophore for antitubercular agents, the activity and the cytotoxicity of which could be improved and reduced with appropriate modification [16]. Introducing a phenylsulfonyl moiety in some molecules may increase the solubility of the molecules and trigger antitumor activity [17,18,19].

Herein, we present a new series of paeonol derivatives combined with the aminothiazole ring as the core structure and further conjugated with the phenylsulfonyl side-chains. With arylsulfonamidothiazole scaffold decoration, the anticancer activity of paeonol may be enhanced through additional hydrogen bonding interactions while retaining or even improving the solubility of paeonol itself [20,21,22]. This new series of aminothiazole-paeonol derivatives was determined to have potential anticancer effects in human gastric adenocarcinoma (AGS), human cervix adenocarcinoma (HeLa), human pancreas adenocarcinoma (PaTu8988t), human colorectal adenocarcinoma (HT-29), human glioblastoma (U87-MG), human lung adenocarcinoma (A549) and mouse colon carcinoma (CT26.WT) cells. Simultaneously, the toxicity of aminothiazole-paeonol derivatives against normal cells was evaluated by embryonic fibroblast (BALB/3T3) cells. The newly synthesized compounds could be structural templates for designing and developing novel anticancer agents.

## 2. Results and Discussion

### 2.1. Chemistry

**Scheme 1:**
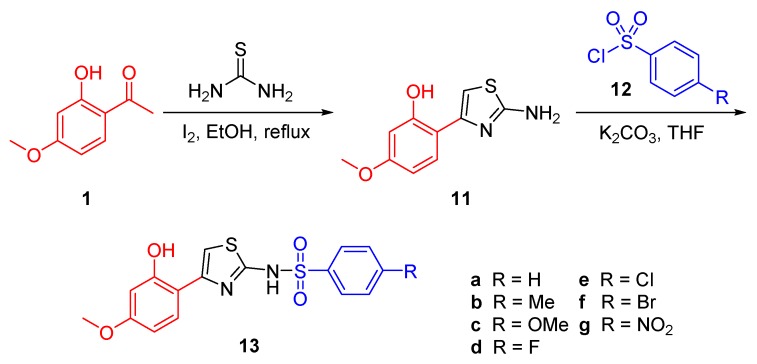
Synthesis of the aminothiazole-paeonol derivatives.

The synthetic methods of preparing the paeonol-2-aminothiazole-phenylsulfonyl derivatives are outlined in Scheme 1. The 2-aminothiazole scaffold was obtained by treating paeonol with thiourea and iodine; the condensation-cyclization of thiourea initiated by iodine afforded compound **11**. To produce various paeonol-phenylsulfonyl derivatives, we treated 2-aminothiazole-paeonol **11** with substituted phenylsulfonyl chloride **12** to yield the final desired compounds **13**. All these products were obtained in sufficient yield and purified by using recrystallization for anticancer assays.

### 2.2. Anticancer Activity and Structure Activity Relationship Analysis

The antitumor effects of the new synthesized compounds against AGS, HeLa, PaTu8988t, HT-29, U87-MG, A549, CT26.WT and BALB/3T3 are described in Table 1. Our results indicated that the aminothiazole-paeonol derivatives exhibited cytotoxic effects toward the tested human cancer cell lines. We observed that compound **13c** was the most potent compound, with IC_50_ values of 4.0 μM to AGS, 4.4 μM to HT-29, 5.8 μM to HeLa, 10.0 μM to CT26.WT, 15.8 μM to PaTu8988t and 22.5 μM to U87-MG. Compound **13c** was the only one providing efficient IC_50_ (less than 50 μM) against U87-MG glioblastoma. Additionally, compound **13c** was relatively less toxic to BALB/3T3 (IC_50_: 32.7 μM) in comparison to 5-FU against BALB/3T3 (IC_50_: 1.0 μM). Compound **13d** was the second most potent compound, showing IC_50_ values of 7.2, 11.2, 13.8 and 31.4 µM to AGS, HT-29, HeLa and PaTu8988t, respectively. However, compound **13d** possessed lower water solubility than compound **13c** did (1.55 *vs.* 3.04 mmol/L, shown in Table 2), which arose from the F and OCH_3_ groups at the *para*-positions of the phenylsulfonyl terminals. On the other hand, compound **13g** was the most inactive compound. In the HT-29 treatment, the activity of the parent compound **13a** was between those of the most potent compound **13c** and the most inactive compound **13g**. Comparing the structures of **13c** and **13g**, compound **13c** was substituted with the OCH_3_ group and compound **13g** was replaced with the NO_2_ group at the *para*-positions of the phenylsulfonyl terminals. In general, the NO_2_ group manifest electron-withdrawing and lipophilic properties in the structure, which could conceivably alter the pharmacokinetic behaviors of compounds. Moreover, the electron-donating OCH_3_ contributed an additional oxygen atom to the structure, making it more hydrophilic and thus increasing the water solubility. Some pharmacokinetic properties are listed in Table 2 [23,24]. All the compounds meet Lipinski’s Rule of Five, with the molecular weights under 500 and the log *p*-values lower than five. In summary, compound **13c** presented the most potent activity against AGS and HT-29 cells with IC_50_ values of 4.0 and 4.4 µM, respectively, which was superior to 5-FU (IC_50_ values: 43.8 and 7.2 µM against AGS and HT-29). The compound **13c** was less toxic against fibroblast cells than 5-FU; therefore, compound **13c** might be a promising lead compound for developing an anticancer agent against gastrointestinal tract–related adenocarcinoma.

**Table 1:** Cytotoxicity of compounds toward various cell lines.

Compounds	IC_50_ (μM)							
	BALB/3T3	AGS	HeLa	PaTu8988t	HT-29	U87-MG	A549	CT26.WT
**13a**	>50	>50	>50	31.1	20.7	>50	>50	>50
**13b**	>50	22.0	27.4	38.7	14.1	>50	>50	>50
**13c**	32.7	4.0	5.8	15.8	4.4	22.5	>50	10.0
**13d**	>50	7.2	13.8	31.4	11.2	>50	>50	>50
**13e**	>50	> 50	>50	>50	13.4	>50	>50	>50
**13f**	>50	> 50	16.4	22.8	11.0	>50	>50	>50
**13g**	32.2	> 50	>50	>50	47.8	>50	>50	30.0
5-Fu *	1.0	43.8	2.2	12.5	7.2	>50	>50	9.2
5-FU ** [25,26,27,28,29,30,31,32]	13.0	79.5	0.232	11.3	19.3	4.9	10.3	61.0
	72 h	24 h	48 h	48 h	48 h	48 h	48 h	72 h

*: the IC_50_ values were measured at 48 h after cells being treated with 5-FU in our study; **: the IC_50_ values of 5-FU against different cancer cells published in other references were measured at 24, 48 or 72 h after treatment.

**Table 2:** Calculation of lipophilicity and water solubility of paeonol derivatives library [23,24].

Compounds	Molecular Weight, Lipophilicity and Water Solubility			
	M.W.	*c*logP	*c*logS	S (mmol/L)
**13a**	362.418	3.26	−4.14	2.625486
**13b**	376.445	3.57	−4.31	1.843748
**13c**	392.444	3.27	−4.11	3.046335
**13d**	380.4804	3.76	−4.39	1.550002
**13e**	396.86	3.83	−4.43	1.474475
**13f**	441.314	4.07	−4.57	1.187812
**13g**	407.026	3.21	−4.33	1.903804

## 3. Experimental Section

### 3.1. General Experimental Procedures

All reactions were carried out in oven-dried glassware (120 °C) under an atmosphere of nitrogen unless indicated otherwise. Dichloromethane, ethanol, ethyl acetate, hexanes, methanol, and THF were purchased from Mallinckrodt Chemical Co. Ethyl acetate was dried and distilled from CaH_2_. Tetrahydrofuran was dried by distillation from sodium and benzophenone under an atmosphere of nitrogen. Benzenesulfonyl chloride, 4-bromobenzenesulfonyl chloride, 4-chlorobenzenesulfonyl chloride, 4-fluorobenzenesulfonyl chloride, 4-methoxybenzenesulfonyl chloride, 4-nitrobenzenesulfonyl chloride, peaonol (2′-hydroxy-4-methoxyacetophenone), potassium carbonate, and *p*-toluenesulfonyl chloride were purchased from Sigma-Aldrich China Inc., Shanghi, China.

Melting points were obtained with a Fargo MP-2D melting point apparatus (melting point range up to ~400 °C). Analytical thin layer chromatography (TLC) was performed on precoated plates (silica gel 60 F-254), purchased from Merck Inc. Infrared (IR) spectra were measured on a Perkin-Elmer Model Spectrum 100 spectrophotometer. Absorption intensities are recorded by the following abbreviations: s = strong; m = medium; and w = weak. Proton NMR spectra were obtained on a Varian Mercury-400 (400 MHz) spectrometer or Bruker AC-400 (400 MHz) spectrometer by use of chloroform-*d* (CDCl_3_) and dimethylsulfoxide-*d*_6_ (DMSO-*d*_6_) as the solvents. Proton NMR chemical shifts were referenced to residual protonated solvents (δ 7.24 for chloroform and δ 2.49 for dimethylsulfoxide). Carbon-13 NMR spectra were obtained on a Varian Mercury-400 (100 MHz) spectrometer by use of chloroform-*d* (CDCl_3_) and dimethylsulfoxide-*d*_6_ (DMSO-*d*_6_) as the solvents. Carbon-13 chemical shifts are referenced to the center of the CDCl_3_ triplet (δ 77.0 ppm) and DMSO septet (δ 39.5 ppm). Multiplicities are recorded by the following abbreviations: s, singlet; d, doublet; t, triplet; q, quartet; m, multiplet; *J*, coupling constant (hertz). High-resolution mass spectra were obtained by means of a JEOL JMS-700 mass spectrometer.

### 3.2. Procedure for the Preparation of Aminothiazole-Paeonol (***11***)

To obtain aminothiazole-paeonol (**11**), paeonol (**1**) (1.0 equiv) was reacted with iodine (1.1 equiv) and thiourea (3.0 equiv) in ethanol under reflux condition for 12.0–16.0 h. Then, the reaction mixture was quenched with NaOH_(aq)_ (2.0 equiv) and the ethanol was removed under reduced pressure. The residue was extracted with ethyl acetate and the combined organic layer were washed the brine and dried over MgSO_4(s)_. After being filtered and condensed under reduced pressure, the crude product was purified by column chromatography on silica gel (ethyl acetate and hexane as eluent) to give compound **11**.

*2-(2-Aminothiazol-5-yl)-5-methoxyphenol* (**11**): ^1^H-NMR (CDCl_3_, 400 MHz): δ 7.40 (d, *J* = 8.4 Hz, 1 H, H-3), 6.54 (s, 1 H, CH), 6.47 (s, 1 H, H-6), 6.42 (dd, *J* = 8.4, 2.0 Hz, 1 H, H-4), 5.05 (s, 2 H, NH_2_), 3.78 (s, 3 H, OMe) ppm. ^13^C-NMR (CDCl_3_, 100 MHz): δ 166.8, 161.0, 157.3, 148.9, 126.6, 111.0, 106.8, 101.6, 98.9, 55.2 (OMe) ppm; HRMS (ESI+) *m*/*z* [M + H]^+^ calculated for C_16_H_14_N_2_O_4_S_2_, 223.0463, found 223.0462.

### 3.3. Standard Procedure for the Preparation of Aminothiazole-Paeonol Derivatives (***13***)

To a solution containing aminothiazole-paeonol (**11**, 1.0 equiv) in anhydrous THF (2.0–3.0 mL) was added potassium carbonate (1.3 equiv) and a sulfonyl chloride **12** (1.1 equiv). After the reaction mixture was stirred at 25 °C for 2.0–3.0 h, it was diluted with dichloromethane (5.0 mL). Inorganic solids were filtered off and the filtrate was concentrated under reduced pressure to afford the residue. It was then purified by use of column chromatography on silica gel (various ratio of methanol to dichloromethane) to give the desired conjugates **13**.

*N-*[4-(2-Hydroxy-4-methoxyphenyl)thiazol-2-yl]*benzenesulfonamide* (**13a**): Yield 80%, green solid product. IR (film): ν 3569.1, 2811.2, 1560.1, 1481.2, 1374.2, 1131.6, 853.4 cm^−1^. ^1^H-NMR (CDCl_3_, 400 MHz): δ 7.58–7.55 (m, 2 H, 2 × ArH), 7.53–7.51 (m, 1 H, H-6), 7.43–7.33 (m, 3 H, 3 × ArH), 6.84 (d, *J* = 2.0 Hz, 1 H, H-3), 6.79–6.77 (m, 1 H, H-5), 6.55 (s, 1 H, SCH), 3.75 (s, 3 H, OMe) ppm. ^13^C NMR (CDCl_3_, 100 MHz): δ 166.8, 163.9, 158.3, 147.2, 143.7, 131.5, 125.9, 121.1, 113.6, 113.2, 108.8, 106.2, 55.6 (OMe) ppm; HRMS (ESI+) *m*/*z* [M + H]^+^ calculated for C_16_H_14_N_2_O_4_S_2_, 363.0395, found 363.0396.

*N-*[4-(2-Hydroxy-4-methoxyphenyl)thiazol-2-yl]*-4-methylbenzenesulfonamide* (**13b**): Yield 84%, off-white solid product. IR (film): ν 3579.1, 2921.2, 1580.1, 1491.2, 1384.1, 1141.6, 843.4 cm^−1^. ^1^H-NMR (CDCl_3_, 400 MHz): δ 7.44 (d, *J* = 8.8 Hz, 1 H, H-6), 7.39 (d, *J* = 8.2 Hz, 2 H, 2 × ArH), 7.06 (d, *J* = 8.2 Hz, 2 H, 2 × ArH), 6.81 (d, *J* = 2.4 Hz, 1 H, H-3), 6.74 (dd, *J* = 8.8, 2.4 Hz, 1 H, H-5) 6.52 (s, 1 H, SCH), 3.72 (s, 3 H, OMe), 2.29 (s, 3 H, CH_3_) ppm. ^13^C-NMR (CDCl_3_, 100 MHz): δ 167.8, 159.3, 147.0, 145.3, 144.4, 132.1, 130.5, 129.1, 128.2, 120.9, 113.1, 108.7, 105.9, 55.4 (OMe), 21.4 (CH_3_) ppm; HRMS (ESI+) *m*/*z* [M + H]^+^ calculated for C_17_H_16_N_2_O_4_S_2_, 377.0551, found 377.0551.

*N-*[4-(2-Hydroxy-4-methoxyphenyl)thiazol-2-yl]*-4-methoxybenzenesulfonamide* (**13c**): Yield 83%, off-white solid product. IR (film): ν 3672.1, 2931.1, 1560.6, 1473.2, 1388.1, 1142.7, 817.4 cm^−1^. ^1^H-NMR (CDCl_3_, 400 MHz): δ 7.46–7.42 (m, 3 H, H-6 + 2 × ArH), 6.86 (d, *J* = 2.8 Hz, 1 H, H-3), 6.76–6.72 (m, 3 H, H-5 + 2 × ArH), 6.57 (s, 1 H, SCH), 3.75 (s, 3 H, OMe), 3.74 (s, 3 H, OMe) ppm. ^13^C-NMR (CDCl_3_, 100 MHz): δ 166.8, 163.9, 159.4, 147.1, 144.7, 130.5, 125.9, 121.0, 113.7, 113.2, 108.8, 106.2, 55.6 (OMe), 55.5 (OMe) ppm; HRMS (ESI+) *m*/*z* [M + H]^+^ calculated for C_17_H_16_N_2_O_5_S_2_, 393.0501, found 393.0498.

*4-Fluoro-N-*[4-(2-hydroxy-4-methoxyphenyl)thiazol-2-yl]*benzenesulfonamide* (**13d**): Yield 81%, white solid product. IR (film): ν 3695.5, 2943.3, 1589.7, 1493.9, 1378.3, 1157.7, 837.7 cm^−1^. ^1^H-NMR (CDCl_3_, 400 MHz): δ 7.51–7.48 (m, 2 H, 2 × ArH), 7.41 (d, *J* = 8.8 Hz, 1 H, H-6), 6.95–6.91 (m, 2 H, 2 × ArH), 6.86 (d, *J* = 2.6 Hz, 1 H, H-3), 6.75 (dd, *J* = 8.8, 2.6 Hz, 1 H, H-5), 6.46 (s, 1 H, SCH), 3.74 (s, 3 H, OMe) ppm. ^13^C NMR (CDCl_3_, 100 MHz): δ 167.3, 167.1, 164.5, 159.4, 146.8, 144.5, 131.2, 130.6, 120.9, 115.9, 115.6, 113.3, 108.9, 105.8, 55.5 (OMe) ppm; HRMS (ESI+) *m*/*z* [M + H]^+^ calculated for C_16_H_13_FN_2_O_4_S_2_, 381.0301, found 381.0302.

*4-Chloro-N-*[4-(2-hydroxy-4-methoxyphenyl)thiazol-2-yl]*benzenesulfonamide* (**13e**): Yield 81%, white solid product. IR (film): ν 3708.8, 2796.7, 1566.74, 1459.55, 1314.15, 1256.54, 984.95 cm^−1^. ^1^H-NMR (CDCl_3_, 400 MHz): δ 7.53 (d, *J* = 8.8 Hz, 1 H, H-6), 7.48–7.46 (m, 2 H, 2 × ArH), 7.29–7.27 (m, 2 H, 2 × ArH), 6.94 (d, *J* = 2.4 Hz, 1 H, H-3), 6.82 (dd, *J* = 8.8, 2.4 Hz, 1 H, H-5), 6.56 (s, 1 H, SCH), 3.81 (s, 3 H, OMe) ppm. ^13^C-NMR (CDCl_3_, 100 MHz): δ 167.2, 159.5, 146.8, 144.7, 140.7, 133.2, 130.7, 129.6, 128.7, 120.9, 113.5, 109.1, 106.0, 55.6 (OMe) ppm; HRMS (ESI+) *m*/*z* [M + H]^+^ calculated for C_16_H_13_ClN_2_O_4_S_2_, 397.0005, found 397.0006.

*4-Bromo-N-*[4-(2-hydroxy-4-methoxyphenyl)thiazol-2-yl]*benzenesulfonamide* (**13f**): Yield 83%, white solid product. IR (film): ν 3691.2, 2838.7, 1591.3, 1479.1, 1362.1, 1143.7, 821.7 cm^−1^. ^1^H-NMR (CDCl_3_, 400 MHz): δ 7.36–7.33 (m, 3 H, H-5 + 2 × ArH), 7.28–7.26 (m, 2 H, 2 × ArH), 6.80 (d, *J* = 1.6 Hz, 1 H, H-3), 6.71 (dd, *J* = 8.8, 1.6 Hz, 1 H, H-5), 6.35 (s, 1 H, SCH), 3.69 (s, 3 H, OMe) ppm. ^13^C-NMR (CDCl_3_, 100 MHz): δ 167.2, 164.3, 158.4, 145.7, 143.6, 132.3, 121.2, 115.6, 113.2, 108.7, 105.2, 56.4 (OMe) ppm; HRMS (ESI+) *m*/*z* [M + H]^+^ calculated for C_16_H_13_BrN_2_O_4_S_2_, 440.9500, found 440.9502.

*N-*[4-(2-Hydroxy-4-methoxyphenyl)thiazol-2-yl]*-4-nitrobenzenesulfonamide* (**13g**): Yield 84%, orange solid product. IR (film): ν 3607.7, 2685.6, 1554.65, 1360.65, 1325.25, 1267.54, 964.95 cm^−1^. ^1^H-NMR (CDCl_3_, 400 MHz): δ 8.06 (d, *J* = 8.0 Hz, 2 H, 2 × ArH), 7.59 (d, *J* = 8.0 Hz, 2 H, 2 × ArH), 7.29 (d, *J* = 8.6 Hz, 1 H, H-6), 6.90 (s, 1 H, H-3), 6.77 (d, *J* = 8.6 Hz, 1 H, H-5), 6.32 (s, 1 H, SCH), 3.76 (s, 3 H, OMe) ppm. ^13^C-NMR (CDCl_3_, 100 MHz): δ 167.2, 162.0, 156.2, 151.1, 147.8, 145.9, 132.9, 128.2, 124.2, 112.8, 107.4, 105.0, 104.2, 55.8 (OMe) ppm; HRMS (ESI+) *m*/*z* [M + H]^+^ calculated for C_16_H_13_N_3_O_6_S_2_, 408.0246, found 408.0247.

### 3.4. Cell Culture

Cell lines, including human gastric adenocarcinoma (AGS), human cervix adenocarcinoma (HeLa), human pancreas adenocarcinoma (PaTu8988t), human colorectal adenocarcinoma (HT-29), human glioblastoma (U87-MG), human lung adenocarcinoma (A549), mouse colon carcinoma (CT26.WT) cells and embryonic fibroblast (BALB/3T3) were purchased from Bioresource Collection and Research Center (BCRC, Taiwan) to evaluate anticancer activity of aminothiazole-paeonol derivatives. Five cell lines were cultured in different media from Gibco (Life Technologies, Grand Island, NY, USA) and Hyclone (GE Healthcare Life Science). Cells were cultured in 90% medium mixed with 10% serum at 37 °C in humidified atmosphere with 5% CO_2_ and grown in T-75 flask with a feeding cycle of two to three days. The composition and procedures of preparing culture media for different cell lines were followed by instruction manual of suppliers [5,33].

### 3.5. Drug Treatment and Cell Viability Assay

The different cell lines were seeded into 96-well tissue culture plates at a concentration of 5 × 10^3^ cells/100 µL/well overnight. Subsequently, the cells were treated with serial concentrations of seven paeonol derivatives. After 48 h of incubation, cell viability of each cell line was determined by MTT colorimetric assay. Briefly, 100 µL of 2 mg/mL MTT reagent (Sigma-Aldrich, St. Louis, MO, USA) was added to each well and incubated for 4 h at 37 °C. Later, the medium was aspirated and 100 µL of dimethyl sulphoxide (DMSO) added to each well; finally, the OD_595_ of each well was measured by ELISA reader (TECAN, Wien, Austria).

## 4. Conclusions

Various phenylsulfonyl side-chains were directly conjugated to paeonol, the major component of a traditional Chinese medicine, Moutan cortex, through chemical synthesis to generate a new series of aminothiazole-paeonol derivatives. The substituents on the phenylsulfonyl side-chain included F, Cl, Br, NO_2_, Me, and OMe. All the synthesized compounds were characterized using ^1^H-NMR, ^13^C-NMR, and mass spectra data. The cytotoxic effects of all compounds were evaluated against fibroblast cells (BALB/3T3) and seven cancer cell lines, including AGS, HeLa, PaTu8988t, HT-29, U87-MG, A549 and CT26.WT. We observed that the thiazole-paeonol-phenylsulfonyl derivatives demonstrated cytotoxic effects against the tested cancer cell lines. We also discussed the structural activity relationship on the basis of screening results and pharmacokinetic properties. Results indicated that **13c** exhibited the most potent activity against AGS and HT-29 cells with IC_50_ values of 4.0 and 4.4 µM, respectively, which is superior to 5-FU (IC_50_ values: 43.8 and 7.2 µM against AGS and HT-29) and may be a promising lead compound to develop an anticancer agent for gastrointestinal tract–related adenocarcinoma.
